# SARS-CoV-2 vaccination influence in the development of long-COVID clinical phenotypes

**DOI:** 10.1017/S0950268825000093

**Published:** 2025-02-04

**Authors:** Patrizia Pasculli, Michele Antonacci, Maria Antonella Zingaropoli, Federica Dominelli, Federica Ciccone, Francesco Pandolfi, Yann Collins Fosso Ngangue, Giorgio Maria Masci, Roberta Campagna, Franco Iafrate, Valeria Panebianco, Carlo Catalano, Ombretta Turriziani, Gioacchino Galardo, Paolo Palange, Claudio Maria Mastroianni, Maria Rosa Ciardi

**Affiliations:** 1 Department of Public Health and Infectious Diseases, Sapienza University of Rome, Rome, Italy; 2 Department of Radiological, Oncological and Pathological Sciences, Policlinico Umberto I, Sapienza University of Rome, Rome, Italy; 3 Department of Molecular Medicine, Sapienza University of Rome, Rome, Italy; 4 Medical Emergency Unit, Sapienza University of Rome, Policlinico Umberto I, Rome, Italy

**Keywords:** anti-S antibodies, COVID-19, mRNA vaccine, PASC, post-COVID-19 syndrome

## Abstract

Although SARS-CoV-2 vaccination reduces hospitalization and mortality, its long-term impact on Long-COVID remains to be elucidated. The aim of the study was to evaluate the different development of Long-COVID clinical phenotypes according to the vaccination status of patients. Clinical and demographic characteristics were assessed for each patient, while Long-COVID symptoms were self-reported and later stratified into distinct clinical phenotypes. Vaccination was significantly associated with the avoidance of hospitalization, less invasive respiratory support, and less alterations of cardiopulmonary functions, as well as reduced lasting lung parenchymal damage. However, no association between vaccination status and the development of at least one Long-COVID symptom was found. Nevertheless, clinical phenotypes were differently associated with vaccination status, as neuropsychiatric were more frequent in unvaccinated patients and cardiorespiratory symptoms were reported mostly in vaccinated patients. Different progression of disease could be at play in the different development of specific Long-COVID clinical phenotypes, as shown by the different serological responses between unvaccinated and vaccinated patients. A higher anti-Spike (*S*) antibody titre was protective for vaccinated patients, while it was detrimental for unvaccinated patients. A better understanding of the mechanism underlying the development of Long-COVID symptoms might be reached by standardized methodologies and symptom classification.

## Introduction

Since the COVID-19 pandemic was officially declared, almost 780 million cases and nearly 7 million deaths have been reported worldwide [1]. Although the acute manifestations of the disease are well characterized and most patients recover within a few weeks, some patients may exhibit long-term effects weeks or months after SARS-CoV-2 infection, regardless of whether they have been admitted to the hospital [2]. This condition is commonly referred to as “Long-COVID”, “post-acute COVID-19 sequelae” (PACS), or “post-acute COVID-19 condition” (PCC), but its definition varies depending on country and institution [3, 4]. Indeed, the World Health Organization (WHO) defines the onset of Long-COVID as occurring 3 months after recovery, whereas the US Centers for Disease Control and Prevention (CDC) puts the onset of ‘Post-COVID Conditions’ at 4 weeks after the acute phase. On the other hand, the British National Institute for Health and Care Excellence (NICE) defines “post-COVID-19 syndrome” as the effects that persist for 12 or more weeks after onset [3].

The term “Long-COVID” is used to describe a condition characterized by a wide range of symptoms that may persist for months or even years after the acute phase of COVID-19. This multisystem condition is known to affect multiple organ systems, and its symptoms can vary considerably from person to person. The most commonly reported symptoms are asthenia, dyspnoea, taste and smell alteration, cardiovascular symptoms, and neurocognitive impairment [4, 5]. Alterations in spirometry parameters, lung capacity, and diffusion capacity for carbon monoxide have been shown to be the most frequent, with COVID-19 survivors exhibiting radiological and functional lung sequelae lasting for 6–12 months after recovery [6]. The British Thoracic Society (BTS) has recommended follow-up of patients with a clinical-radiological diagnosis of COVID-19 pneumonia [7]. Particularly, patients with severe pneumonia should undergo pulmonary function tests (PFTs) 12 weeks after discharge; meanwhile, in patients with only mild to moderate pneumonia, PFTs must be conducted only after abnormal chest radiography [7].

Development of Long-COVID symptoms appears to be influenced by a variety of factors, such as gender, age, body mass index (BMI), the need for hospitalization or intensive care unit (ICU) admission, vaccination status, and the presence of comorbidities (e.g. diabetes mellitus and hypertension) [8]. However, Long-COVID has been observed to develop in all of the spectrum of COVID-19 disease, ranging from asymptomatic to severe illness [9, 10].

As of 31 December 2023, the WHO estimated that 56% of the general population had completed a primary series of vaccination, of which 28% had received at least one booster dose of a COVID-19 vaccine [11]. Nevertheless, despite SARS-CoV-2 vaccination reducing the risk of severe symptoms, vaccinated people can still be infected and suffer from asymptomatic, mild, or moderate forms of disease [9, 12]. Post-vaccination infections (i.e. breakthrough cases) are a growing phenomenon and have been shown to be a risk factor for the onset of Long-COVID and its manifestations, including cardiovascular, gastrointestinal, musculoskeletal, and neurologic disorders [9, 13]. Furthermore, despite the correlation between the number of vaccine doses and the prevention of Long-COVID, the upregulation of the immune response triggered by the vaccination and the higher antibody titre is correlated with worse sequelae [9, 14]. However, some studies have indicated that vaccination is associated with a reduction in the incidence and severity of Long-COVID symptoms, decreasing the risk of death in people who experience breakthrough SARS-CoV-2 infections [13]. As the ambiguity regarding the effects of vaccination on Long-COVID symptoms remains, it emphasizes the need for further investigations.

The aim of this study was to compare the different manifestations of Long-COVID clinical phenotypes according to the vaccination status of patients, as well as identify additional risks or protective factors in the development of these symptoms.

## Materials and methods

### Recruitment criteria and stratification

A single-centre retrospective study was conducted on COVID-19 patients who received medical care at the Policlinico Umberto I, Sapienza University of Rome. Inclusion criteria consisted of age > 18 years and a confirmed diagnosis of COVID-19 independently of their anti-SARS-CoV-2 vaccination at follow-up. The exclusion criteria were as follows: patients who had more than one SARS-CoV-2 infection prior to the visit, patients who died, patients who were transferred to nursing homes or assisted living facilities, and patients who refused to undergo follow-up (FU).

Patients were stratified into two populations: unvaccinated and vaccinated, both invited to the Long-COVID clinic to be examined by a multispecialty medical team. Additionally, the anti-SARS-CoV-2 vaccination status was addressed by stratifying vaccinated patients into partially vaccinated, if they had received a partial or completed primary vaccine series (1 or 2 doses), and fully vaccinated, if they had received at least one COVID-19 vaccine booster dose (1 or more booster doses). This was conducted in accordance with the definition of the primary vaccine series for SARS-CoV-2, as reported by the majority of European countries in the strategies and deployment plans proposed by the European Centre for Disease Prevention and Control [15].

A further stratification of the study population according to hospitalization during the acute stage of COVID-19 was performed. Two groups were created: in-patients, who required hospitalization during the acute stage of COVID-19, and out-patients, who managed the disease at home. The extent of the required respiratory support (oxygen/ventilation) needed during hospitalization was classified accordingly as no oxygen support needed (ambient air, AA), oxygen delivered through conventional O_2_ masks (Ventimask, VMK), non-invasive mechanical ventilatory support (through continuous positive airway pressure (CPAP) or high flow nasal cannula (HFNC), and invasive mechanical ventilatory support in patients admitted to the ICU.

### Data collection of long-COVID symptoms

**Figure 1.**
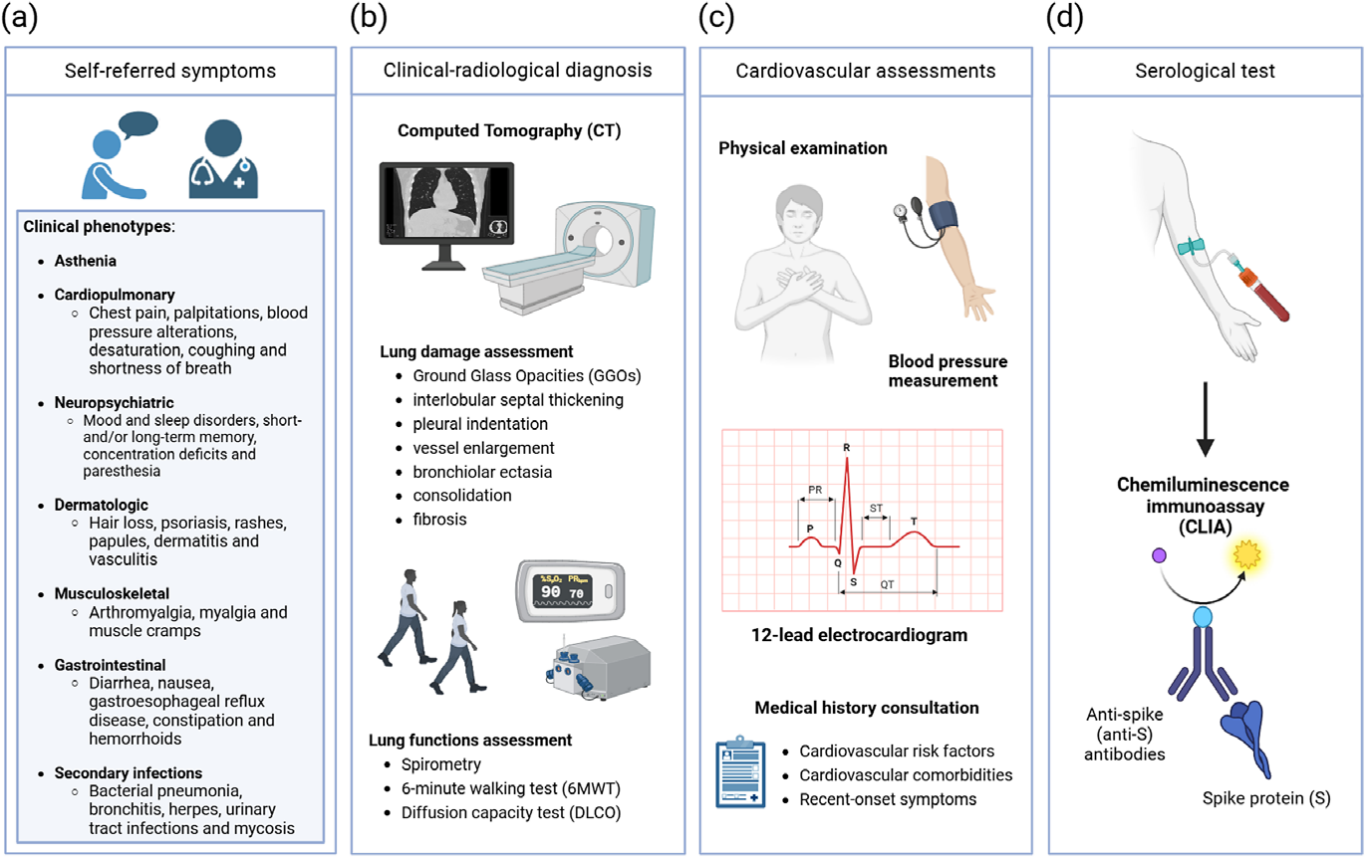
Study population Long-COVID visit (A) Long-COVID symptoms reported during the examination were distributed into distinct clinical phenotypes (B) Clinical-radiological diagnosis of pulmonary functions was performed through the combination of spirometry, 6-minute walking test (6MWT) and the use of chest CT (Computed Tomography) attributing to each CT scan a severity scores (CTSS); (C) Cardiology visit and assessment of each patient’s presence of cardiovascular risk factors, cardiovascular comorbidities, and any recent-onset symptoms compatible with long-COVID diagnosis; (D) Evaluation of SARS-CoV-2-specific total anti-Spike (anti-S) IgG antibodies using chemiluminescence immunoassay (CLIA).

At least 3 months after the acute phase of SARS-CoV-2 infection, all patients with a confirmed diagnosis of COVID-19 were contacted by telephone and invited to attend an active follow-up visit at a Long-COVID clinic, as recommended by the National Institute for Health and Care Excellence (NICE) Guidelines [16]. At this follow-up visit, patients who had consented to participate in the program were asked to self-report any symptoms associated with Long-COVID to a health professional. The symptoms were recorded irrespective of their actual manifestation and subsequently categorized into different clinical phenotypes based on previous studies [17–22], as illustrated in Figure 1A. Furthermore, patients were examined by a multidisciplinary medical team, which performed pulmonary function and cardiovascular assessments, as well as serological testing (Figure 1B–1D) .

**Figure 2.**
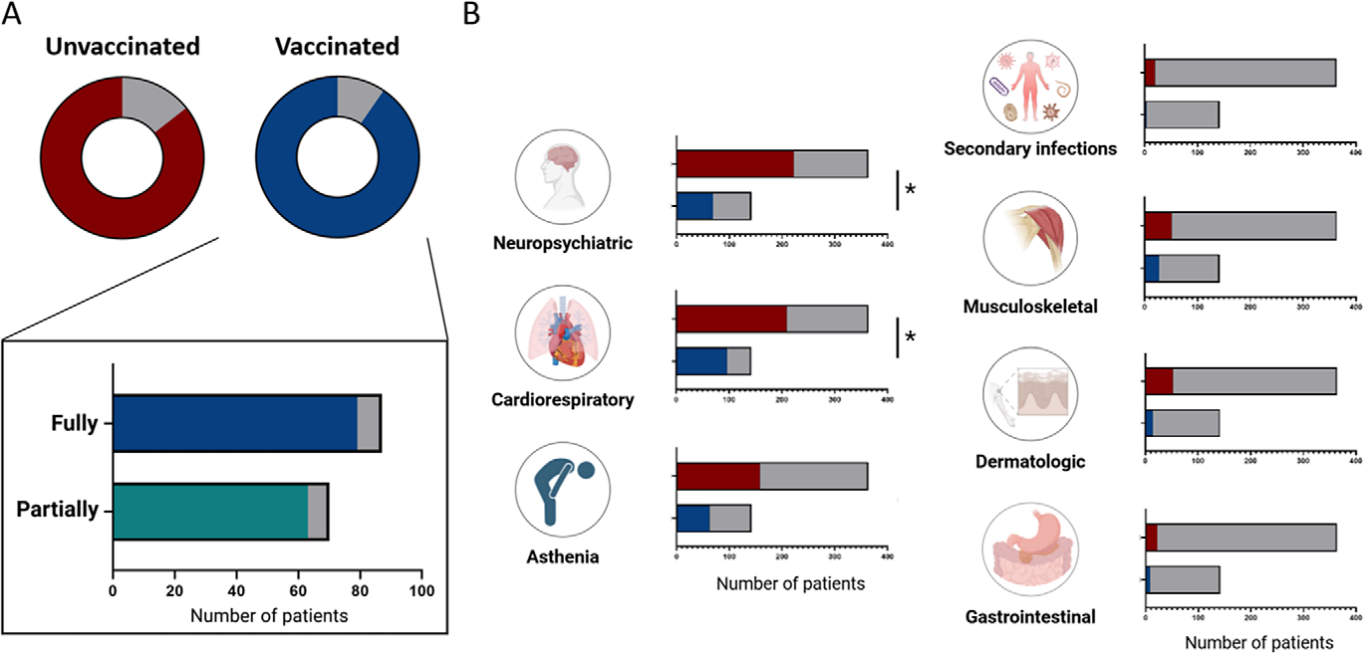
(A) Long-COVID symptoms in unvaccinated and vaccinated patients, stratified for vaccine doses. The percentages of patients with and without Long-COVID symptoms are reported in blue/red and grey, respectively (B) Frequency of unvaccinated (red) and vaccinated (blue) patients self-reported cluster of symptoms during Long-COVID evaluation. Patients who did not report any symptoms are indicated by the color grey. *: 0.05 < p < 0.01.

Clinical-radiological assessment of pulmonary functions was conducted using spirometry and the 6 min walking test (6MWT), in combination with the use of Somatom Sensation 16 and Somatom Sensation 64 multidetector CT (computed tomography) scanners (Siemens Healthineers, Germany). Reconstruction of 1 mm slice thickness images was obtained with the classic filtered back-projection method with a soft tissue kernel of B20 and a lung kernel of B60 (Figure 1B) [25].

Patients who experienced COVID-19 pneumonia in the acute phase underwent a chest CT. A radiologist assessed the damage, in accordance with the standard glossary for thoracic imaging reported by the Fleischner Society [23], through examination of lung parenchymal imaging findings, attributing to each chest CT a CT severity score (CTSS) to indicate the extent of anatomic involvement [24, 25].

Cardiovascular assessments via medical history, blood pressure measurement, 12-lead electrocardiogram, and physical examination were conducted, identifying each patient’s presence of cardiovascular risk factors, cardiovascular comorbidities, and recent-onset symptoms compatible with Long-COVID diagnosis (Figure 1C).

Serological tests were performed using a commercial chemiluminescence immunoassay (CLIA) (DiaSorin LIAISON SARS-CoV-2 TrimericS IgG; DiaSorin S.p.A, Italy) for the detection of SARS-CoV-2-specific total anti-Spike (anti-*S*) IgG antibodies (Figure 1D).

### Statistical analyses

All data are reported as median with interquartile range (IQR). Comparative analyses were performed, specifically the chi-square test or Fisher test to compare frequencies of categorical variables or Student’s *t*-test, Mann–Whitney test, and Kruskal–Wallis, Fisher test of quantitative variables. Results were considered statistically significant if the *p*-value was ≤0.05. All analyses were performed with *R* v4.0.2 [26] and GraphPad Prism v9.2.0.

### Ethics

The study was approved by the Ethics Committee of Policlinico Umberto I, Sapienza University of Rome (protocol number 298/2020). All patients were given written consent.

## Results

### Clinical and demographic characteristics

Between 6 May 2020 and 19 February 2024, 582 COVID-19 patients were enrolled for this follow-up study (272 females and 310 males), with a median age (IQR of 58 [49–66] years) (Table 2, Figure 2).

**Figure 3.**
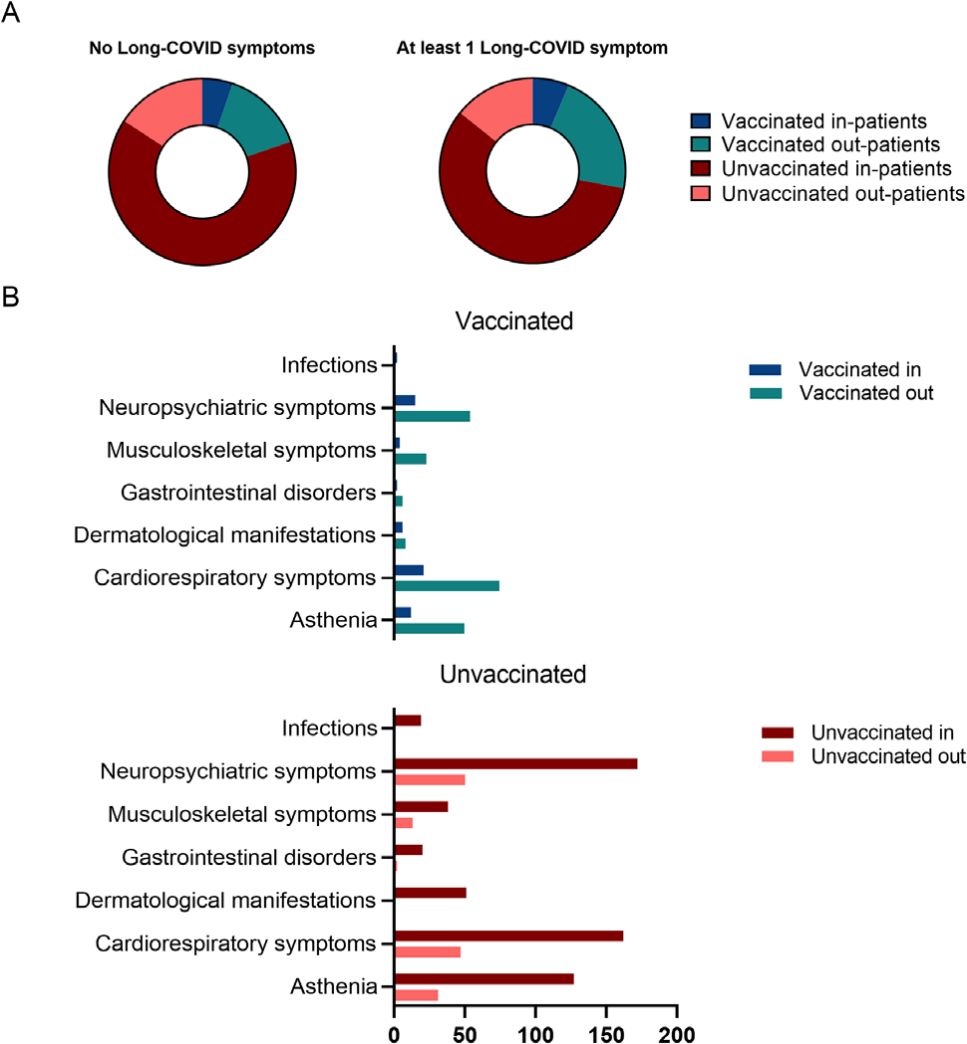
(A) Association between hospitalization and development of Long-COVID symptoms in vaccinated and unvaccinated patients (B) Hospitalization and self-reported cluster of symptoms in vaccinated and unvaccinated patients.

Patients were stratified into two groups: unvaccinated and vaccinated. The unvaccinated group consisted of 425 individuals. Conversely, the vaccinated group included 157 patients who underwent vaccination before SARS-COV-2 infection, 70 (44.6%) of whom were partially vaccinated and 87 (55.4%) were fully vaccinated (Table 1).

**Table 1.** Demographic and clinical characteristics of the study population stratified according to vaccination status

	Unvaccinated	Vaccinated
**Female**	184/425 (43%)	88/157 (56%)
**Male**	241/425 (57%)	69/157 (44%)
**Age (years), median (IQR)**	58 (49–66)	57 (45–65)
**Hospitalization**		
In-patients	341/425 (80%)	36/157 (23%)
Out-patients	84/425 (20%)	121/157 (77%)
**Respiratory support**		
AA	160/425 (37.6%)	131/157 (83.4%)
VMK	152/425 (35.8%)	16/157 (10.2%)
CPAP/HFNC	94/425 (22.1%)	10/157 (6.4%)
ICU	19/425 (4.5%)	0/157 (0%)
**Vaccination cycle**		
Partially completed (1 or 2 doses)	0/425 (0%)	70/157 (44.6%)
Fully completed (3 or more doses)	0/425 (0%)	87/157 (55.4%)

*Note:* IQR: interquartile range; AA: ambient air; VMK: ventimask; CPAP/HNFC: continuous positive airway pressure/high flow nasal cannula; ICU: intensive care unit.

**Figure 4.**
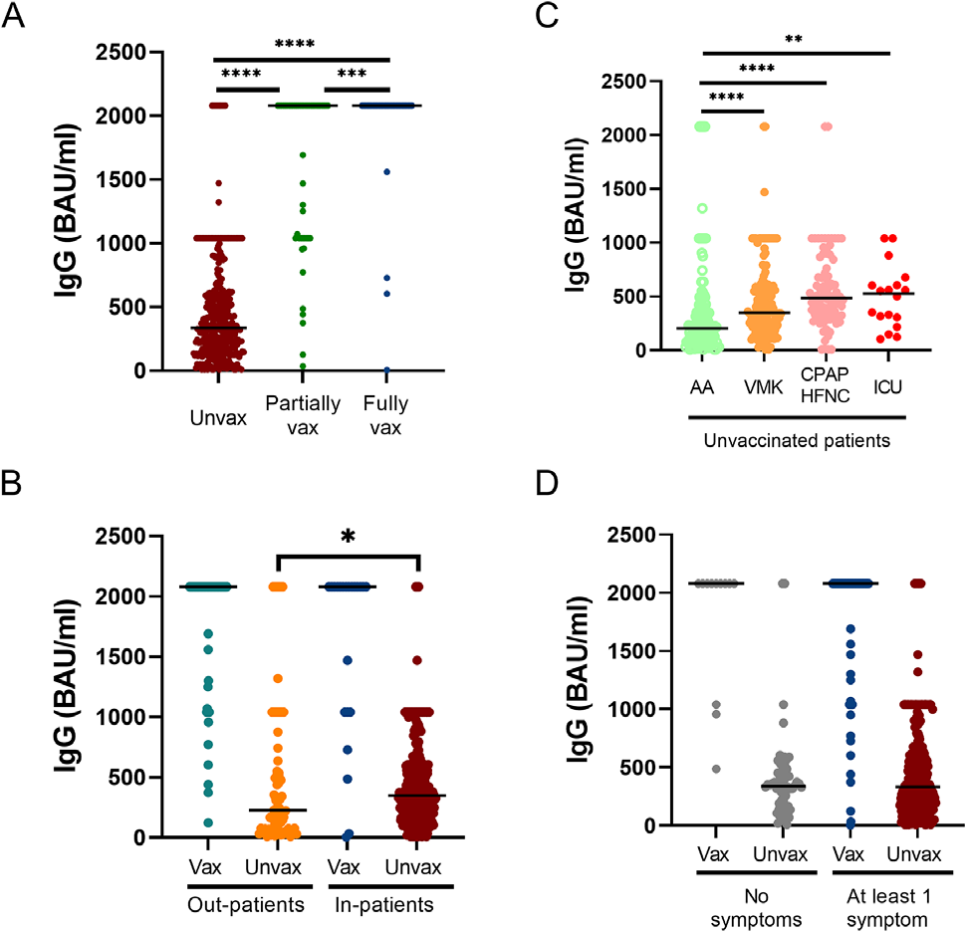
(A) Anti-Spike (S) IgG antibody titer measured in fully vaccinated, partially vaccinated and unvaccinated patients (B) Different serological response based on hospitalization status during acute phase of COVID-19 of vaccinated and unvaccinated patients (C) Anti-S IgG antibody titer variations in differently treated patients of the unvaccinated group during acute phase of COVID-19 (D) Correlation between serological response of vaccinated and unvaccinated patients and development of Long-COVID symptoms. Ns: p>0.05; *: 0.05 < p < 0.01; **: 0.01 < p < 0.001; ***: 0.001 < p < 0.0001; ****: p > 0.0001; Vax: Vaccinated.

No difference in age between the unvaccinated and vaccinated groups was observed. Conversely, a higher vaccination rate among the female subgroup compared to the male subgroup was observed (56% vs. 44%, respectively, *p* = 0.0067) (Table 1).

At least one symptom was reported by 506 patients. No statistical significance between vaccination status and development of at least one Long-COVID symptom was found (Table 1 and Figure 2A), regardless of whether patients were partially or fully vaccinated (90% vs. 91%). Coherently, the two populations of patients did not show any significant difference in the development of asthenia (43.6% vs. 43.4%), secondary infections (2.1% vs. 5.2%), and symptoms of the dermatological (9.9% vs. 14.3%), gastrointestinal (5.6% vs. 6%), and musculoskeletal clusters (19% vs. 14%) (Figure 2B). Cardiorespiratory symptoms were mostly observed in vaccinated patients (67.6% vs. 57.4%, *p* = 0.0430), while neuropsychiatric disorders were more frequent in unvaccinated ones (48.6% vs. 61%, *p* = 0.0124) (Figure 2B).

CTSS was reported for 372 (89%) of the patients who underwent chest CT (Table 2). Cardiovascular assessment showed significantly more alterations in the unvaccinated group compared to the vaccinated group (70.3% vs. 55.4%, p = 0.0464) (Table 2). Furthermore, clinical-radiological assessment of pulmonary damage revealed a higher incidence of both spirometry (31.3% vs. 79.6%, p < 0.0001) and chest CT alterations (34.2% vs. 69.6%, p < 0.0001), as well as more pronounced pulmonary damage (p < 0.0001), among unvaccinated patients (Table 2). Indeed, the extent of respiratory support required was different, as the vaccinated group underwent a less invasive treatment compared to the unvaccinated patients (Table 2). No statistical difference between full and partial vaccination was observed (AA: 75.7% vs. 89.7%; VMK: 14.3% vs. 6.9%; CPAP/HFNC: 10% vs. 3.5%).

**Table 2.** Long-COVID clinical characteristics stratified according to vaccination status

	Unvaccinated	Vaccinated
**Long-COVID symptoms**		
Any	364/425 (86%)	142/157 (90%)
Asthenia	158/364 (43.4%)	62/142 (43.7%)
Cardiorespiratory	209/364 (57.4%)	96/142 (67.6%)
Dermatological	52/364 (14.3%)	14/142 (10%)
Gastrointestinal	22/364 (6%)	8/142 (5.6%)
Musculoskeletal	51/364 (14%)	27/142 (19%)
Neuropsychiatric	222/364 (61%)	69/142 (48.6%)
Secondary infections	19/364 (5.2%)	3/142 (2.1%)
**Long-COVID alterations**		
CT scan alterations	208/299 (69.6%)	25/73 (34.2%)
CTSS	3 (0–8)	0 (0–1)
Spirometry alterations	168/211 (79.6%)	47/150 (31.3%)
Cardiovascular alterations	52/74 (70.3%)	62/112 (55.4%)

*Note:* IQR: interquartile range; COVID: coronavirus disease; CT: chest tomography; CTSS: chest tomography severity score.

### Out-patients and in-patients

Overall, 373 patients were hospitalized during the acute phase of infection (in-patient) while 209 were managed at home (out-patient) (Table 2). Age and male sex were observed as risk factors for hospitalization (*p* < 0.0001 and *p* < 0.0001, respectively) (Table 3). After stratification based on vaccination status, age was confirmed as a risk factor for hospitalization in both the unvaccinated and the vaccinated groups (*p* < 0.0001 and *p* = 0.0176, respectively) (Table 3). Conversely, male sex was associated with a higher rate of hospitalization in unvaccinated patients only (87.1% vs. 71.2%, *p* < 0.0001) (Table 3).

**Table 3.** Demographic and clinical characteristics of the study population stratified according to vaccination status and hospitalization

	Unvaccinated (=425)		Vaccinated (=157)	
	Out-patients (n = 84)	In-patients (n = 341)	Out-patients (n = 121)	In-patients (n = 36)
**Female**	53/84 (63%)	131/341 (38.4%)	72/121 (59.5%)	16/36 (44.4%)
**Male**	31/84 (37%)	210/341 (61.6%)	49/121 (40.5%)	20/36 (55.6%)
**Age (years), median (IQR)**	53 (41–62)	59 (51–68)	57 (43–65)	59 (53–71)
**Vaccination cycle**				
Partially completed (1 or 2 doses)	0/84 (0%)	0/341 (0%)	48/121 (39.7%)	22/36 (61.1%)
Fully completed (3 or more doses)	0/84 (0%)	0/341 (0%)	73/121 (60.3%)	14/36 (38.9%)
**Respiratory support**				
AA	84/84 (100%)	76/341 (22.3%)	121/121 (100%)	10/36 (27.8%)
VMK	0/84 (0%)	152/341 (44.6%)	0/121 (0%)	16/36 (44.4%)
CPAP/HFNC	0/84 (0%)	94/341 (27.6%)	0/121 (0%)	10/36 (27.8%)
ICU	0/84 (0%)	19/341 (5.5%)	0/121 (0%)	0/36 (0%)

*Note:* IQR: interquartile range; AA: ambient air; VMK: ventimask; CPAP/HNFC: continuous positive airway pressure/high flow nasal cannula; ICU: intensive care unit.

A lower rate of hospitalized vaccinated patients compared to out-patients was observed (*p* < 0.0001), especially in fully vaccinated ones (*p* = 0.0348) (Table 3). Furthermore, unvaccinated and partially vaccinated patients were hospitalized significantly longer when compared to fully vaccinated patients (19 [11–28], 13.5 [10–20], and 7 [0–18], respectively; *p* = 0.0016).

Hospitalization did not influence the development of at least one Long-COVID symptom (Figure 3A), even after stratification with vaccination status (Table 3). However, clinical phenotypes reported depended on whether vaccinated and unvaccinated patients were hospitalized or not. Neuropsychiatric symptoms had a higher prevalence among out-patients of the unvaccinated group compared to their vaccinated counterparts (69.4% vs. 49.1%, *p* = 0.0090) (Table 3 and Figure 3B). Additionally, unvaccinated patients exhibited a higher number of dermatological manifestations (17.5% vs. 1.4%, *p* = 0.0001) and secondary infections (6.51% vs. 0%, *p* = 0.0184) when hospitalized (Table 3 and Figure 3B). Asthenia and symptoms of the cardiorespiratory, gastrointestinal, and musculoskeletal clusters were not associated with hospitalization in either group of patients (Table 3 and Figure 3B).

During Long-COVID evaluation, hospitalized patients showed a higher number of pulmonary alterations, identified by spirometry (75.9% vs. 40.4%, *p* < 0.0001) and chest CT (73.2% vs. 34.9%, *p* < 0.0001), and a larger extent of pulmonary damage, determined by a worse CTSS (*p* < 0.0001) (Table 4). In-patients were also characterized by more cardiovascular alterations compared to out-patients (54.4% vs. 45.6%, *p* = 0.0005) (Table 4). Vaccinated patients who managed the disease at home were less likely to develop cardiovascular alterations compared to hospitalized vaccinated patients (47.6% vs. 78.6%, *p* = 0.0046) (Table 4), while spirometry alterations were significantly exacerbated in hospitalized unvaccinated patients compared to out-patients (83.4% vs. 66.7%, respectively; *p* = 0.0148) (Table 4). In addition, hospitalization was associated with increased pulmonary alterations, both in unvaccinated patients (73.1% vs. 52%, *p* = 0.0042) and vaccinated (75% vs. 18.9%, *p* < 0.0001), with a higher CTSS in unvaccinated patients in both the in- and out- subgroups (in-patients: 4 [0–8] in unvaccinated and 1 [0.3–1] in vaccinated, *p* = 0.0002; out-patients: 4 [0–8.8] in unvaccinated and 0 [0–0] in vaccinated, *p* < 0.0001) (Table 4). Consistently, both unvaccinated and vaccinated groups required more invasive respiratory support when hospitalized (*p* < 0.0001) (Table 4). No significant difference was observed when comparing respiratory support required by hospitalized patients of the unvaccinated and vaccinated groups (Table 4).

**Table 4.** Long-COVID clinical characteristics stratified according to vaccination status and hospitalization

	Unvaccinated (=425)		Vaccinated (=157)	
	Out-patients (n = 84)	In-patients (n = 341)	Out-patients (n = 121)	In-patients (n = 36)
**Long-COVID symptoms**				
Any	72/84 (85.7%)	292/341 (85.6%)	110/121 (90.9%)	32/36 (88.9%)
Asthenia	31/72 (43%)	127/292 (43.5%)	50/110 (45.4%)	12/32 (37.5%)
Cardiorespiratory	47/72 (65.3%)	162/292 (55.5%)	75/110 (68.2%)	21/32 (65.6%)
Dermatological	1/72 (1.4%)	51/292 (17.5%)	8/110 (72.7%)	6/32 (18.7%)
Gastrointestinal	2/72 (2.8%)	20/292 (68.5%)	6/110 (54.5%)	2/32 (6.2%)
Musculoskeletal	13/72 (18%)	38/292 (13%)	23/110 (20.9%)	4/32 (12.5%)
Neuropsychiatric	50/72 (69.4%)	172/292 (58.9%)	54/110 (49.1%)	15/32 (46.9%)
Secondary infections	0/72 (0%)	19/292 (6.5%)	1/110 (0.9%)	2/32 (6.2%)
**Long-COVID alterations**				
CT scan alterations	26/50 (52%)	182/249 (73.1%)	10/53 (18.9%)	15/20 (75%)
CTSS	1 (0–6)	4 (0–8)	0 (0–0)	1 (0.3–1)
Spirometry alterations	32/48 (66.7%)	136/163 (83.4%)	35/118 (29.7%)	12/32 (37.5%)
Cardiovascular alterations	12/20 (60%)	40/54 (74.1%)	40/84 (47.6%)	22/28 (78.6%)

*Note:* IQR: interquartile range; COVID: coronavirus disease; CT: chest tomography; CTSS: chest tomography severity score.

### Serological response in long-COVID

The serological response was measured in 482 (82.8%) patients. A higher IgG antibody titre in vaccinated patients compared to unvaccinated ones was observed (2080 [2080–2080] and 335 [173–581], respectively; p < 0.0001), particularly in those patients who completed the vaccination cycle (2080 [1040–2080] partially vaccinated and 2080 [2080–2080] fully vaccinated; p = 0.0001) (Figure 4A).

Out-patients of the unvaccinated group showed a lower serological response compared to in-patients (227 [74–616] and 348 [203–581], respectively, p = 0.0447) (Figure 4B). Besides, unvaccinated patients revealed a higher serological response when they received any kind of respiratory support compared to patients who did not need it (AA: 206 [113–390], VMK: 351 [222–585], CPAP/HFNC: 486.2 [329–742], ICU: 528 [285–624]; p < 0.0001) (Figure 4C). No difference was observed in the anti-S IgG titre between out- and in-patients of the vaccinated group (out-patients: 2080 [2080–2080]; in-patients: 2080 [1040–2080]) (Figure 4B).

Finally, no statistical significance was found between anti-S IgG titre and the development of at least one Long-COVID symptom (368 [218–588] for 0 symptoms and 447 [208–1040] for at least 1 symptom), neither when stratified in unvaccinated (339 [166–459] for 0 symptoms and 333 [174–603] for at least 1 symptom) nor vaccinated patients (2080 [1300–2080] for 0 symptoms and 2080 [1625–2080] for at least 1 symptom) (Figure 4D).

## Discussion

Given the variety of symptoms and the lack of comprehensive understanding of the underlying mechanisms, the role of vaccination in developing Long-COVID symptoms remains a topic of ongoing debate and controversy [27]. Contrary to consensus, our results showed no statistically significant difference in the occurrence of at least one Long-COVID symptom between unvaccinated and vaccinated individuals, irrespective of whether patients had received partial or complete vaccination [28]. This may instead be attributed to patient-specific factors, such as underlying health conditions and/or comorbidities [29]. Nevertheless, clinical phenotypes developed were differently associated with vaccination status and hospitalization. The observed heterogeneity in the manifestation of Long-COVID clinical phenotypes may reflect the different progression of the disease in the respective patient groups [30]. The unvaccinated cohort exhibited a significant prevalence of neuropsychiatric symptoms, dermatological manifestations, and secondary infections. Hospitalization during the acute phase of SARS-CoV-2 infection is recognized as a predisposing factor for the development of neuropsychiatric symptoms among Long-COVID patients [17, 31]. Additionally, the persistence of SARS-CoV-2 RNA in brain tissue biopsies months or even years after acute COVID-19 [32, 33] has been associated with the development of Long-COVID symptoms [34]. Together with the lower viral clearance observed in unvaccinated patients [35], it might explain the higher frequency of neuropsychiatric symptoms in this group. On the other hand, dermatological manifestations and secondary infections experienced by hospitalized unvaccinated patients may be of nosocomial origin, likely due to the heightened immune dysregulation observed in these individuals following the acute phase of SARS-CoV-2 infection [36, 37]. Conversely, cardiorespiratory symptoms were more frequently reported in vaccinated patients, independent of hospitalization. These symptoms, often reported by Long-COVID patients [38], may result from a chronic inflammatory response evoked by persistent viral reservoirs in the heart following the acute infection [39]. Cardiorespiratory symptoms may also be linked to an autoimmune response to cardiac antigens through molecular mimicry [40], which, in turn, might be caused by the evasion of the protective role of SARS-CoV-2 vaccination [39]. Indeed, immunization is associated with an increase in cardiovascular adverse effects [41]. However, the period of infection and the variant of concern (VOC) could play a role in the different pathophysiology of disease experienced by unvaccinated and vaccinated patients [42].

In line with other studies, vaccination has been shown to significantly reduce the risk of hospitalization, with vaccine effectiveness (VE) increasing proportionally to the number of doses received. This effect is evident in both reduced ICU admissions and shorter durations of time spent under medical care [43, 44]. Additionally, vaccination was found to be protective against hospitalization in male patients, as unvaccinated patients of the same sex exhibited a higher rate of hospital admissions [45]. Nevertheless, age was independently associated with a higher risk of hospitalization regardless of vaccination status [45].

The protective role of SARS-CoV-2 vaccination in preventing hospital admission and severe disease is further supported by evidence of fewer residual cardiologic and chest CT abnormalities, as well as milder lung involvement (CTSS), in vaccinated patients than unvaccinated ones [44]. These findings may be attributed to the increased serological response observed with booster doses, which has been associated with a milder clinical presentation of the disease [44, 46]. Consistently, fully vaccinated patients exhibited higher anti-S IgG levels in comparison to partially vaccinated and unvaccinated patients [47]. However, a more pronounced serological response was observed in unvaccinated patients who required hospitalization and respiratory support of any kind compared to those who managed the disease at home. These findings contrast with previous evidence suggesting that higher anti-S IgG levels serve as a protective factor against invasive mechanical intervention [47]. Nonetheless, these levels of anti-S IgG antibodies were still lower than those found in both the vaccinated out-patient and in-patient groups. These results suggest that a more controlled immune activation was achieved when patients were immunized before infection, which may have contributed to the mitigation of the dysregulated systemic inflammatory response [35, 48].

While our study provides valuable insights into the association between vaccination and the development of Long-COVID, certain limitations must be acknowledged. This single-centred retrospective study included a small sample size of vaccinated patients and people younger than 18 years old were not included, reducing generalizability. Besides, the notable discrepancy between patients with recurrent and/or recent Long-COVID symptoms compared to those who did not experience any symptoms may be attributed to the fact that the latter may be less inclined to undergo follow-up, which could potentially influence the results. Additionally, symptoms were self-referred by the patients and might not be uniform and accurate. The symptoms were also mainly classified into clinical phenotypes, and the prevalence of individual symptoms was not reported (except for asthenia). A further potential limitation is the lack of data available for the pulmonary, cardiovascular, and serological assessments of some patients. Finally, our findings could be influenced by the different periods of infection.

This retrospective observational study emphasizes the significant impact of SARS-CoV-2 vaccination in reducing the incidence of hospitalization, the need for mechanical ventilation, and the prevalence of cardiopulmonary damage. However, the available data do not indicate that vaccination prior to infection provides protection against the development of Long-COVID. The different manifestations of Long-COVID clinical phenotypes in both unvaccinated and vaccinated patients reveal the complex relationship between vaccination status, the severity of acute COVID-19 disease, and the subsequent onset of Long-COVID symptoms. Further research employing standardized methodologies and symptom classification is essential to improve our understanding of the mechanism underlying the various aetiologies and the effectiveness of vaccines in preventing them.

## Data Availability

All data generated or analysed during this study are included in this published article. The datasets used and/or analysed during the current study are available from the corresponding author upon reasonable request.
